# Comparative Study of Catalytic Activity of Recyclable Au/Fe_3_O_4_ Microparticles for Reduction Of 2,4‐Dinitrophenol and Anionic, Cationic Azo Dyes.

**DOI:** 10.1002/open.202300297

**Published:** 2024-04-16

**Authors:** Ikhbayar Batsukh, Tegshjargal Khishigjargal, Uuriintuya Dembereldorj, Munkhtsetseg Sambuu, Erdene‐Ochir Ganbold, Erdene Norov

**Affiliations:** ^1^ Department of Chemical and Biological Engineering School of Engineering and Applied Sciences National University of Mongolia; ^2^ School of Educational Studies Mongolian National University of Education; ^3^ Department of Physics School of Arts and Sciences National University of Mongolia; ^4^ Institute of Physics and Technology Mongolian Academy of Sciences Ulaanbaatar 13330 Mongolia

**Keywords:** Catalytic reaction, reusable catalyst, Au/Fe_3_O_4_, gold sub-microparticle, magnetic catalyst

## Abstract

We synthesized Au/Fe_3_O_4_ microparticles. Initially, citrate‐capped Fe_3_O_4_ micro‐sized particles were synthesized by the co‐precipitation method with an excess amount of trisodium citrate. Gold ions were reduced on the surface of citrate‐capped Fe_3_O_4_ and grew as gold sub‐microparticles with an average diameter of 210 nm on the surface. The characteristic SPR peak of gold nanoparticles on the surface of Fe_3_O_4_ was detected at 584 nm, whereas the absorption in the near‐infrared region was increased. SEM images has proved that the synthesized Au/Fe_3_O_4_ composite microparticles has an average diameter of 1.7 micrometers. The results of XRD patterns proved the existence of both crystal phases of Fe_3_O_4_ and Au particles. To investigate the catalytic activity, the reaction rate constant of reduction of 2,4‐dinitrophenol (2,4‐DNP) and degradation of Congo red (CR), and methylene blue (MB) with NaBH4 in the presence of Au/Fe_3_O_4_ catalyst was monitored by UV‐Vis spectroscopy. The initial reaction rate constant calculated from the change in characteristic peak absorptions of 2,4‐dinitrophenol was 3.97×10^−3^ s^−1^, while the reaction rate constants for the degradation of CR and MB were 9.72×10^−3^ s^−1^ and 14.25×10^−3^ s^−1^ respectively. After 5 cycles, Au/Fe_3_O_4_ microparticles preserved 99 % of the reaction rate constant, exhibiting considerable recycling efficiency in the reduction of nitro groups.

## Graphical abstract

The current study investigates a comparative analysis of Au/Fe_3_O_4_ microparticles as reusable catalysts for the reduction of three different organic pollutants namely, 2,4‐dinitrophenol, Congo red, and Methylene blue dyes. Results indicate that Au/Fe_3_O_4_ microparticles possess great catalytic activity for the reduction of those pollutants and can be reused for the reduction of 2,4,–dinitrophenol, and Congo red dye even after 5 cycles.

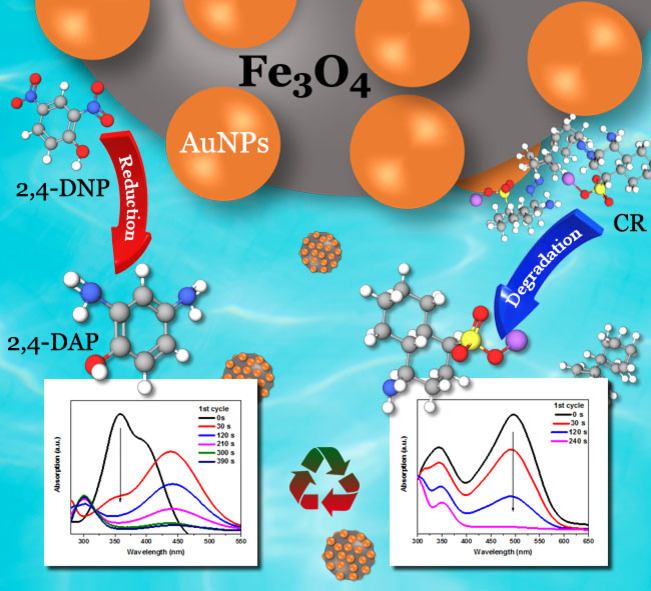

## 1 Introduction

Transition metal nano and sub‐microparticles possess unique physical and chemical properties, attracting great interest from material scientists.[^1^, ^2^] Among them, gold nanoparticle was vastly applied to various fields such as biomedical sensors, detectors, surface‐enhanced Raman scattering (SERS), photothermal therapy, drug delivery, and catalysts.[^3^, ^4^, ^5^, ^6^, ^7^] Most notably, gold nanoparticles demonstrated that they can be the most promising catalyst due to excellent catalytic activity for the reduction of nitro groups and degradation of anionic and cationic organic dyes.[^8^, ^9^] Unfortunately, after a single reduction reaction, massive non‐reversible aggregation takes place due to its high surface energy which causes a decrease in the catalytic activity of gold nanoparticles.[^10^, ^11^] Hence, it is expensive to utilize in large‐scale industrial applications because bare gold nanoparticles can't be used in more than a single catalytic reaction as performed in the initial reaction unless its surface is functionalized.^[12]^ For this reason, a core‐shell and supported gold catalyst structures consisting of gold particles or nano‐sized gold shells linked to the Fe_3_O_4_ core are successfully introduced to this field of study.[^13^, ^14^]

For this purpose, it has been reported that using the Au supported on Fe_3_O_4_ nanoparticles for the reduction of p‐nitrophenol and organic dyes can be an efficient reusable catalyst.[^15^, ^16^] This kind of structure prevents Au/Fe_3_O_4_ nano and microparticles from aggregation and can be easily separated from the solution by using an external magnet, making it an economical choice compared to other industrial wastewater treatment methods.^[17]^ Previous studies investigated reusing possibilities and conversion percentages of Au/Fe_3_O_4_ nanoparticles utilizing for mostly reduction of p‐nitrophenol and organic dyes, especially azo dyes. Their results showed that Au/Fe_3_O_4_ nanoparticles converted up to 96.4 % of nitro derivates to amino groups after a 6–8 cycle reaction. It must be indicated that Au/Fe_3_O_4_ nanoparticles possess great reusing ability during the cycle reactions without any major aggregations and reaction rate constant decrement.[^18^, ^19^] Therefore, Au/Fe_3_O_4_ particles became the most desired material for the reduction of organic pollutants in waste water due to its main advantages such as overall stability in dispersed form or reaction mixture, easy surface modification using a variety of functional groups, effective reusability with magnetic separation, and simple synthesizing methods for different structures.^[20]^ Compared to other non‐supported gold and metal nanoparticles, Au/Fe_3_O_4_ nanoparticles possess superior reusing ability towards the reduction of organic pollutants. For example, cobalt nanoclusters were utilized as a reusable catalyst for the reduction of 4‐NP, however, it lost almost half of the catalytic activity only after 5 cycles.^[21]^ Even more, polygonal Au nanoparticles exhibited great catalytic activity for the reduction of 3 different isomeric nitrophenols, unfortunately, its normalized reaction rate was significantly reduced after 5 cycles.^[22]^ In contrast, Au/Fe_3_O_4_ nanoparticles almost can potentially retain their initial reaction rate constant and catalytic activity for up to 8 cycles without significant decrement.^[23]^

To achieve Au/Fe_3_O_4_ magnetic composite materials for highly efficient and stable heterogeneuos catalysts, previous works of literature mentioned many synthesizing methods. Among the numerous synthesizing techniques, one of the simplest approaches is producing the Fe_3_O_4_, first, using the co‐precipitating method and capping by citrate ions to reduce and stabilize and reduce the gold ion on the surface. This approach doesn't require toxic organic precursors, high reaction temperatures, or special requirements.[^24^, ^25^, ^26^] Stein et al.^[27]^ found that citrate‐stabilized Au‐coated Fe_3_O_4_ composite nanoparticles are highly stable in the aqueous solution, for up to 21 days. Its structural stability can remain as a highly dispersed nanoparticle form and be stored for later use. Also, the applications of citrate‐stabilized Au/Fe_3_O_4_ composite nanoparticles in specific fields such as DNA monitoring, photothermal therapy, biological separation, and electrochemical sensors have been widely studied over recent years.[^28^, ^29^, ^30^, ^31^]

This study aimed to investigate the reusable catalytic activity of Au/Fe_3_O_4_ composite microparticle for reduction of the most widespread organic pollutants with 3 different groups such as methylene blue (cationic dye), Congo red (anionic dye) which account for 70 % of all the dyes used in industry and 2,4‐DNP is the essential compound for explosives, synthesis of organic dyes, wood preservatives, plastic manufacturing, pharmaceutical industries, and pesticides.[^32^, ^33^, ^34^] Such organic compounds commonly occur in industrial wastewater, and they are notorious for their high toxicity against living organisms and carcinogen effect.^[35]^ Therefore, reducing the toxicity of water contaminated by those organic pollutants becomes necessary before discharge.^[36]^ Based on these reasons, effective, affordable, recyclable, and stable catalysts with high surface area are required to satisfy such demands.

Despite the numerous studies that reported the reusability and conversion efficiency of Au/Fe_3_O_4_ composite nanoparticles, knowledge about catalytic activity and the ability to reuse the micro‐sized Fe_3_O_4_ support with Au sub‐microparticles, especially for the reduction of 2,4‐dinitrophenol, degradation of Congo red and Methylene blue dyes, is insufficient.[^37^, ^38^, ^39^] Here in, we are reporting the comparative study of the reduction of 3 different functional groups, namely, nitro, azo, and thiazine groups, using micro‐sized Au/Fe_3_O_4_ composite particles as a catalyst. The present study chose 2,4‐dinitrophenol as a model reaction for a compound containing 2 nitro groups. Congo red represents an organic dye consisting of 2 azo groups and methylene blue for the thiazine group also classified as a cationic azo dye. Micro‐sized Au/Fe_3_O_4_ composite particles exhibited great recycling efficiency after 5 cycles on the reduction of 2,4‐dinitrophenol and Congo red dye, in contrast, Au/Fe_3_O_4_ composite particles can not be used more than 1 cycle in the reduction of methylene blue.

## Experiments

### Materials

Hydrogen tetrachloroaurate(III)trihydrate(HAuCl_4_ ⋅ 3H_2_O, 99.9 %) was obtained from Sigma Aldrich, analytical grade ferric chloride (FeCl_3_ ⋅ 6H_2_O), ferrous chloride tetrahydrate (FeCl_2_ ⋅ 4H_2_O) were purchased from Xilong Scientific. Trisodium citrate (Na_3_C_6_H_5_O_7_, ≥99.0 %), sodium hydroxide (NaOH, ≥96.0 %), and sodium borohydride (NaBH_4_, ≥99.0 %) were acquired from Xilong Scientific. Methylene blue (MB, C_16_H_18_ClN_3_S, ≥96.0 %) was obtained from Xilong Scientific. Double‐distilled deionized water is used to prepare solutions. Congo red (CR, C_32_H_22_N_6_Na_2_O_6_S_2_), 2,4‐dinitrophenol (2,4‐DNP, C_6_H_4_N_2_O_5_) were provided by the Institute of Physics and Technology, Institute of Chemistry and Chemical Technology of Mongolian Academy of Science, respectively.

### Preparation of Citrate‐Capped Fe_3_O_4_ Particle

The co‐precipitation method of synthesizing Fe_3_O_4_ particles was modified and adapted from Zhou et al.^[28]^ Generally, a total of 1.62 g of FeCl_3_ ⋅ 6H_2_O and 0.99 g of FeCl_2_ ⋅ 4H_2_O iron salts are dissolved in 40 ml of double‐distilled water and then vigorously stirred until an ionic solution is prepared. To produce magnetic Fe_3_O_4_, 10 ml of 8.25 M NaOH solution is added to the Fe^2+^/Fe^3+^ ionic solution dropwise, as a consequence, the black precipitate is formed. After waiting for 10 minutes, 4.4 g powder trisodium citrate is added to the dark mixture, and maintained the reaction temperature at 90 °C, for an hour. The reaction mixture is cooled down to room temperature then the citrate‐capped Fe_3_O_4_ particle is separated from its supernatant using a board magnet and rinsed with distilled water 3 times. Then it is dispersed to 40 ml deionized water and fully sealed to avoid further oxidation.

### Preparation of Au/Fe_3_O_4_ Composite Microparticle

Initially, 20 ml of 1.27 mM HAuCl_4_ solution is prepared and heated up to 98 °C while continuously stirring. Water‐dispersed citrate‐capped Fe_3_O_4_ particles are sonicated for 30 minutes to prevent agglomeration. After the temperature of the HAuCl_4_ solution reaches 98 °C, 0.3 ml of citrate‐capped Fe_3_O_4_ particle suspension is dropped into the Au^3+^ ionic solution instantly. Then stirring is continued for 45 minutes, finally, the color of the solution changed from black to maroon which indicates the formation of Au/Fe_3_O_4_ composite microparticles. Composite microparticles are separated from the solution by a magnet and rinsed 3 times with distilled water, then dispersed to 1 ml deionized water.

### Characterization of Au/Fe_3_O_4_ Composite Microparticles

Scanning electron microscopy (SEM) is utilized to evaluate the morphology of the Au/Fe_3_O_4_ composite microparticles. Zeta potentials are measured by using dynamic light scattering (DLS) and Fourier transform infrared spectroscopy (FT‐IR SHIMADZU, IR Prestige‐21) is used to detect the vibrations of chemical bonds. The UV‐Vis spectrum of the Au/Fe_3_O_4_ composite microparticles is captured using UV‐Vis spectroscopy (Shimadzu UV‐2410PC) and the crystal structure is characterized by employing X‐ray diffraction analysis (XRD, Maxima‐X7000).

### Catalytic Activity and Reusability of Au/Fe_3_O_4_ Composite Microparticles for 2,4‐DNP by NaBH_4_

The catalytic activity of synthesized Au/Fe_3_O_4_ composite microparticles is examined in a 2 ml quartz cuvette with 1 cm thickness. Before the catalytic activity, 1 ml of NaBH_4_ (1 M) is added to the aqueous mixture consisting of 1 ml of Au/Fe_3_O_4_ composite microparticle and 4.5 mg and 20 ml of 2,4‐DNP (2 mM) solution while constantly stirring. The catalytic reaction is monitored by capturing the absorption spectrum in the range between 250 and 550 nm with UV‐Vis spectroscopy. At the constant time interval, 0.15 ml of the reaction mixture was taken and dispersed in 1.35 ml water in the measurement cuvette to record the catalytic process. The solution color changed from yellow to brownish red over time which indicates the reduction reaction occurring in the presence of the reducing agent NaBH_4_.

### Catalytic Activity and Reusability of Au/Fe_3_O_4_ Composite Microparticles for CR by NaBH_4_

For the degradation of CR, a 2 ml quartz cuvette with 1 cm thickness is utilized. Same as the previous procedure, 1 ml of NaBH_4_ (1.5 M) is added to the mixture consisting of 1 ml of Au/Fe_3_O_4_ microparticle and 4.5 mg and 20 ml of CR (0.8 mM) solution while constantly stirring. The catalytic reaction is monitored by capturing the absorption of light range between 300 and 650 nm with UV‐Vis spectroscopy. At the constant time interval, 0.15 ml of reaction mixture was taken and dispersed in 1.35 ml water in the measurement cuvette to record the catalytic process. The solution color changed from dark red to brownish red over time which indicates the degradation of Congo red.

### Catalytic Activity and Reusability of Au/Fe_3_O_4_ Composite Microparticles for MB by NaBH_4_

The procedure of degradation of methylene blue in the presence of Au/Fe_3_O_4_ microparticle was the same as the reduction of DNP and degradation of Congo red. However, the only difference was the methylene blue solution with 0.2 mM concentration was applied. The blue color of the solution vanished when the addition of 1 ml 0.02 M NaBH_4_ was introduced to the reaction mixture.

## 2 Result and Discussion

### 2.1 Morphological and Structural Analysis of Au/Fe_3_O_4_ Composite Microparticles.

**Figure 1:**
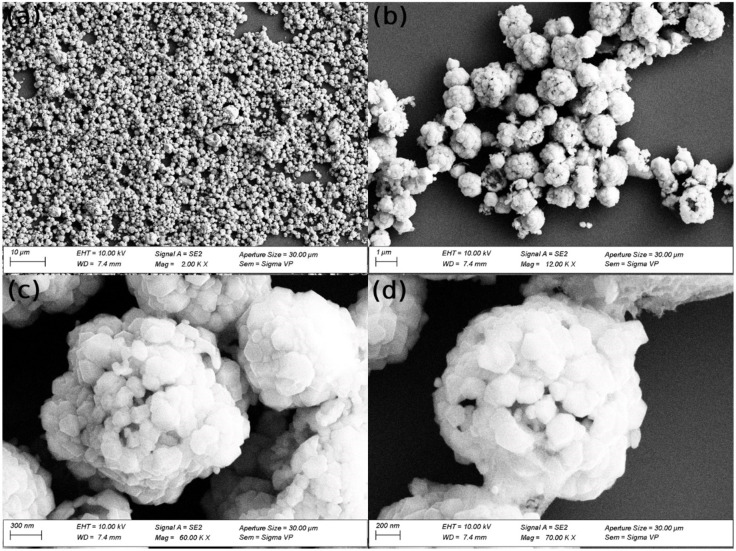
(a–d) SEM images of Au/Fe_3_O_4_ composite microparticles at different magnification scales.

Figure 1 illustrates synthesized Au/Fe_3_O_4_ composite microparticles. The average diameter of the Au/Fe_3_O_4_ composite microparticle was around 1.7 μm and the gold particles located on the surface of the Fe_3_O_4_ particles were about 210 nm as shown in Figure 1a, b. Most of these Au/Fe_3_O_4_ composite microparticles are spherically shaped and possess micro popcorn‐shaped structures. It is obvious that after Fe_3_O_4_ is capped by citrate ions on the surface, gold ions are diffused to the layer of citrate and start to be reduced and stabilized. As a result, the gold nanoparticles were formed. The Au/Fe_3_O_4_ composite microparticle reached its final form once all the Au^+^ ions in the solution were converted and reduced to gold nanoparticles on the surface of the Fe_3_O_4_ microparticles.

**Figure 2:**
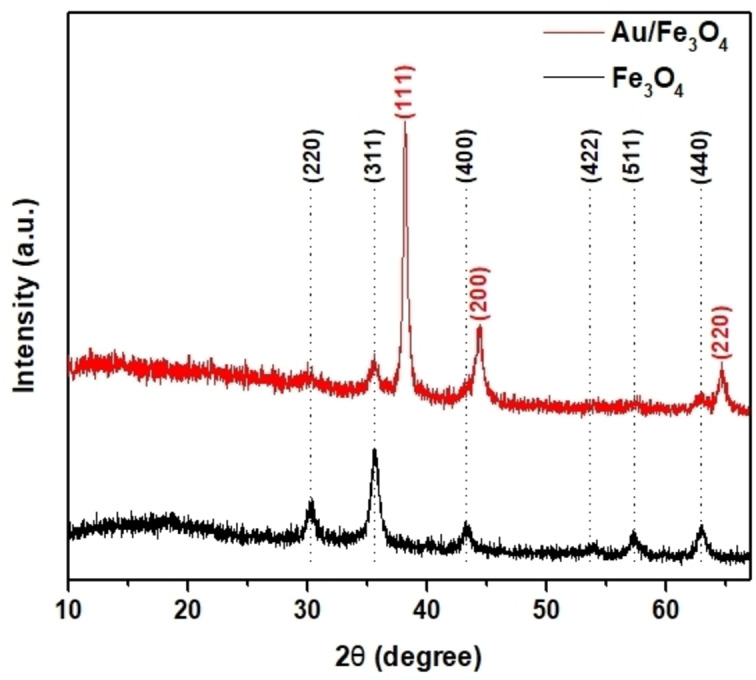
XRD patterns of Au/Fe_3_O_4_ composite microparticles (red) and Fe_3_O_4_ particle (black).

XRD patterns of bare Fe_3_O_4_ particles synthesized by the co‐precipitation method and Au/Fe_3_O_4_ composite microparticles are illustrated in Figure 2. Diffraction peaks were detected at 30.26°, 35.60°, 43.30°, 53.78°, 57.30° and 62.95° can be indexed for diffraction planes of (220), (311), (400), (442), (511) and (440), respectively, of face‐centered cubic spinel structure of Fe_3_O_4_ with fd‐3 m space group. Au/Fe_3_O_4_ composite microparticle showed additional diffraction peaks located at 38.16°, 44.34°, and 64.65° associated with (111), (200), and (220) planes of the face‐centered cubic structure of gold nanoparticles. XRD patterns of Au/Fe_3_O_4_ composite microparticles were consistent with previous studies that revealed XRD patterns of Au/Fe_3_O_4_ structures.[^40^, ^41^] Intensities of gold sub‐microparticles of diffraction peaks were sharper than Fe_3_O_4_ suggesting that gold sub‐microparticles possessed higher crystallization. This result supports the successful fabrication of Au/Fe_3_O_4_ composite microparticles.^[42]^ In other words, two distinct diffraction peaks demonstrate the formation of Au/Fe_3_O_4_ composite microparticles and the successful reduction and nucleation of gold sub‐microparticles on the surface of the Fe_3_O_4_ microparticles. According to the Debye‐Scherrer equation, the average crystallite size of the Au sub‐micro particle was found to be 16.82 nm from the XRD pattern. At the same time, the crystallite size of the Fe_3_O_4_ microparticle was determined to be 11.98 nm which indicates the Fe_3_O_4_ microparticle has lower crystallization than Au sub‐microparticles.

### 2.2 Optical Analysis of Au/Fe_3_O_4_ Composite Microparticles.

**Figure 3:**
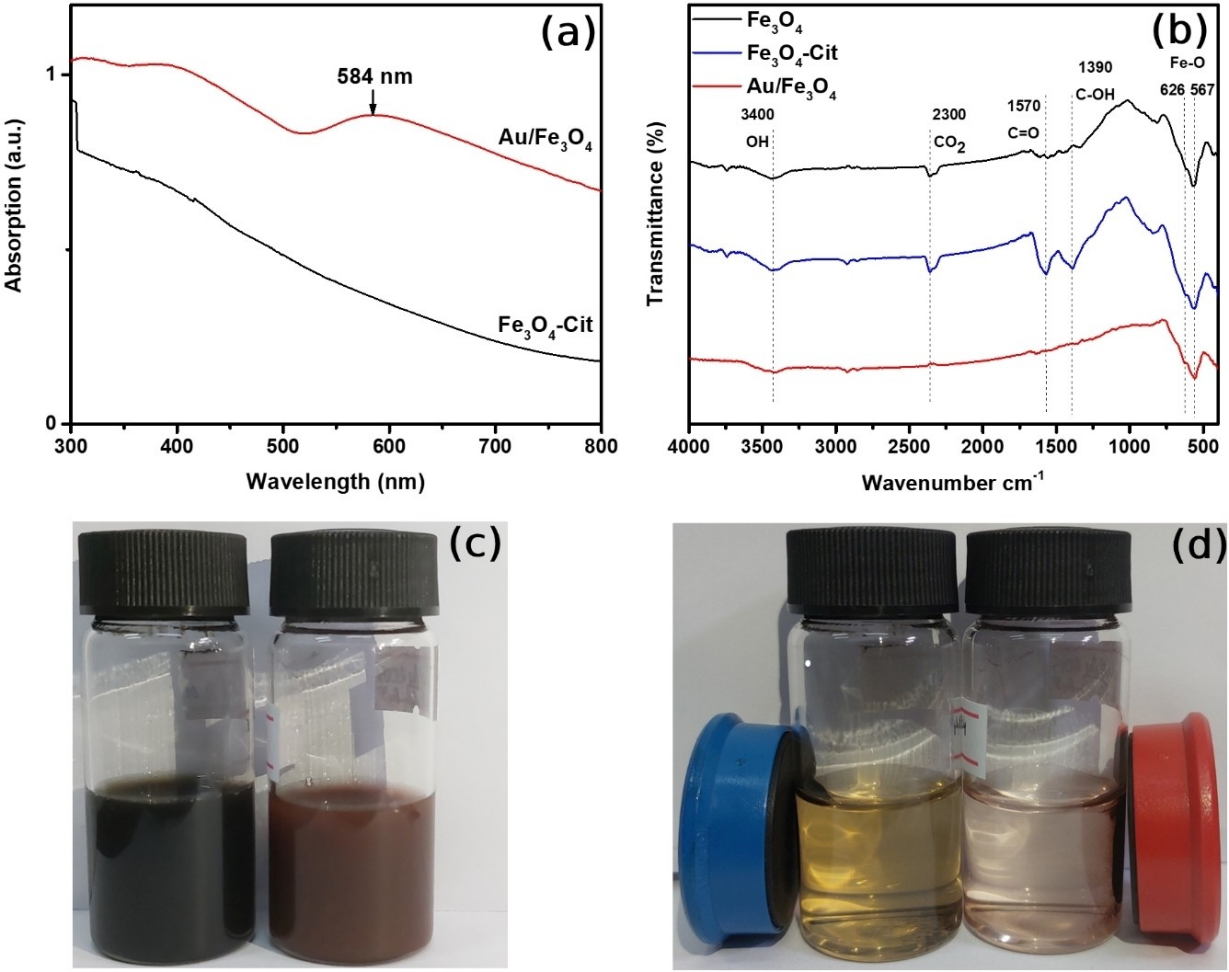
(a) UV‐Vis absorptions, and (b) FT‐IR spectra of synthesized bare and citrate capped Fe_3_O_4_ particles and Au/Fe_3_O_4_ composite microparticles. (c) and (d) represents the photo image of the Fe_3_O_4_ particles and Au/Fe_3_O_4_ composite microparticles dispersed in a water solution and separated by a permanent magnet respectively.

To better understand the effect of Au/Fe_3_O_4_ composite morphology on optical properties, we employed UV‐vis spectroscopy to study the absorption spectra of Au/Fe_3_O_4_ composite microparticles and magnetic Fe_3_O_4_ particles, as shown in Figure 3a. It can be seen from the absorption spectra that Fe_3_O_4_ particles do not have any noticeable peaks in the range between 300 and 800 nm. In contrast, the peak located at 584 nm represents the gold sub‐micro particle's characteristic peak of SPR. Surprisingly, the overall absorption of Au/Fe_3_O_4_ composite microparticle particles was significantly increased compared to bare Fe_3_O_4_ particles suggesting that the optical property of Au/Fe_3_O_4_ composite microparticles can be modified by altering the morphology. It should be noted that the absorption peak associated with the SPR of gold micro and nanoparticles depends on its morphology and size.^[43]^ Therefore, it can be concluded from the broad absorption peak at around 584 nm, that the size distribution of gold sub‐microparticles on the surface of the Fe_3_O_4_ particle was heterogenous which proves the results of the SEM image. In addition, absorption spectra corresponding to gold particles increased in near infra‐red regions which is an indication that Au/Fe_3_O_4_ composite microparticles possess potential for biological applications.[^43^, ^44^] Also, gold particles’ surface plasmon peak can make a redshift as its morphology and composition are changed. The bare gold nanoparticle's peak of SPR is expected to be located at 520 nm. According to Aghamolaei et al.,^[45]^ when the gold‐supported structure is introduced to gold nanoparticles, its plasmon resonance makes a redshift around 570 nm which is consistent with our result, 584 nm. This redshift is associated with the electron transport between Fe_3_O_4_ microparticles and Au sub‐microparticles.^[46]^

FT‐IR spectroscopy results of Fe_3_O_4_ particles, citrate‐capped Fe_3_O_4_ particles, and Au/Fe_3_O_4_ composite microparticles are illustrated in Figure 3b. Absorption bands located at around 567 cm^−1^ and 630 cm^−1^ correspond to Fe−O′s characteristic vibrations, verifying the presence of Fe_3_O_4_ particles. Moreover, stretching of the hydroxyl group is observed at 3400 cm^−1^ due to the negatively charged OH^−^ group being adsorbed on the surface of Fe_3_O_4_ particles during the co‐precipitation synthesizes and this result is repeated at all samples. After the addition of citrate ion, noticeable 2 peaks are detected at 1570 and 1390 cm^−1^ which are related to asymmetric stretching of C=O and symmetric stretching of COO^−^ groups of carboxyl groups respectively. These results also confirmed that the citrate ions capped the surface of Fe_3_O_4_ microparticles by chemisorption process and the carboxylate group formed complex with Fe atoms. Once the gold sub‐microparticle completely grew on the surface of the Fe_3_O_4_ particle, there was notable vibration bands were detected in the given range. However, the intensity of peaks associated with the carboxyl group was lowered because the gold sub‐microparticles were reduced by the citrate ion. Figures 3c and d show the photo image of the Fe_3_O_4_ particles and Au/Fe_3_O_4_ composite microparticles dispersed in water and separated from the solution using the external magnet. It can be clearly seen that the solution of the F_3_O_4_ particles is black, in contrast, Au/Fe_3_O_4_ composite microparticles were colored maroon when it is the colloid solution. Both samples can be easily separated from their solvent by just applying the board magnet which confirms that both samples have magnetic properties.

The measured zeta potentials of synthesized samples are summarized in Table 1. All the synthesized samples were negatively charged. The surface charge of citrate‐capped Fe_3_O_4_ was −77.27 mV due to the high amount of citrate ion attached to the surface. In contrast, gold sub‐microparticles blocked some parts of citrate ions on the surface of the Fe_3_O_4_ particle after the gold particles′ growth. Also, Au^3+^ ions were diffused into the citrate ion layer to be reduced and stabilized by citrate ion, therefore, the zeta potential of Au/Fe_3_O_4_ composite microparticles was significantly reduced compared to citrate‐capped Fe_3_O_4_ and found to be −35.61 mV.

**Table 1:** Zeta potentials of citrate‐capped Fe_3_O_4_ particles and Au/Fe_3_O_4_ composite microparticles.

	Zeta potential (mV)	Error (mV)
**Fe_3_O_4_‐Cit**	−77.27	±4.12
**Au/Fe_3_O_4_**	−35.61	±2.17

### 2.3 Catalytic Activity and Reusability of Au/Fe_3_O_4_ Composite Microparticles.

**Figure 4:**
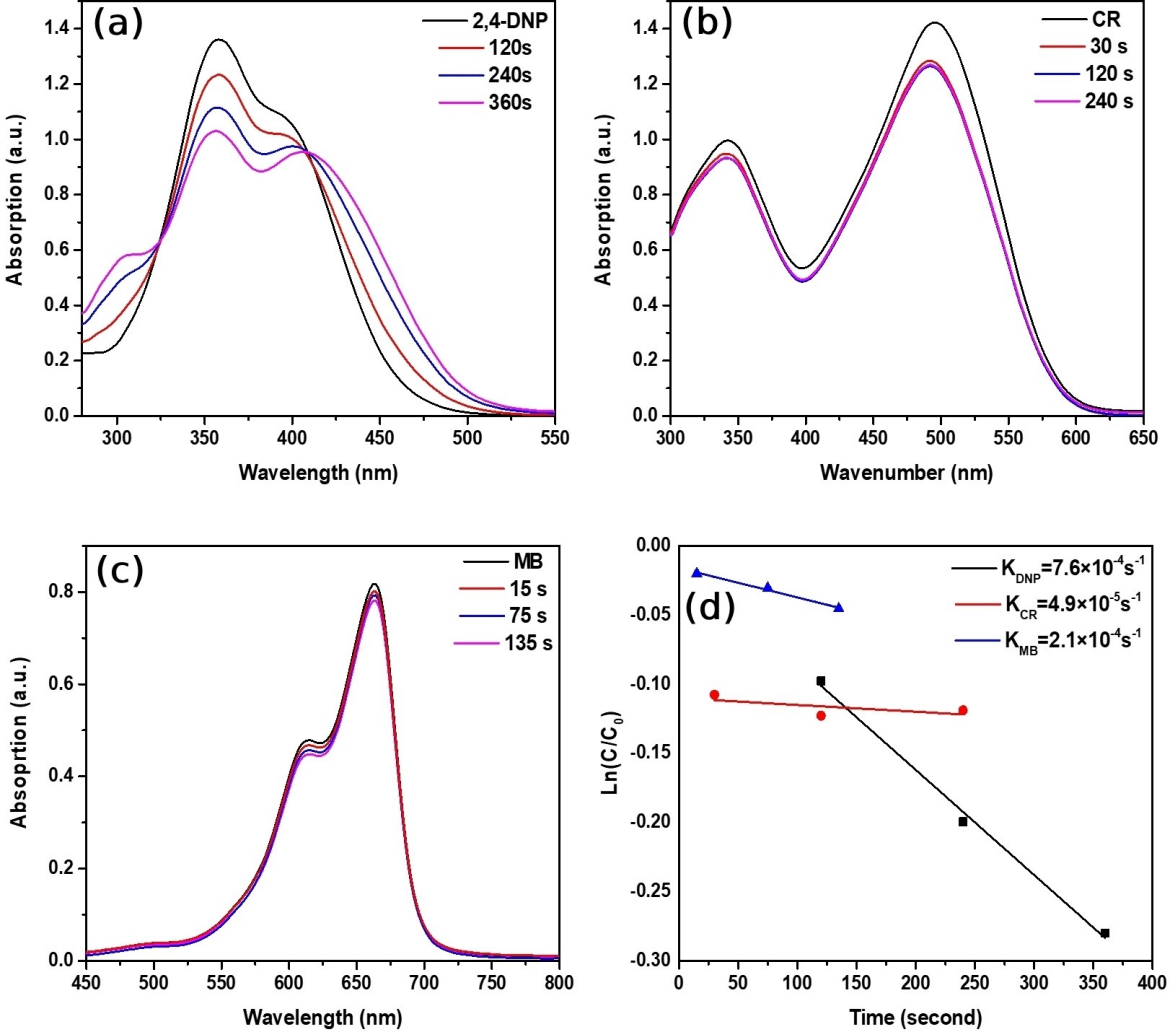
(a, b, c) Control measurement of the reduction of 2,4‐DNP, CR, and MB organic pollutants by NaBH_4_ in the absence of catalyst, (d) First‐order kinetic plots of ln(C/C_0_) vs time.

To investigate rate constants of reduction of 2,4‐DNP, CR, and MB dyes in the absence of a catalyst, the time‐dependent UV‐Vis absorption spectra were shown in Figure 4a–c. Excess amounts of strong reducing agent NaBH_4_ can reduce the 2,4‐DNP, Congo red, and Methylene Blue dyes, therefore, we conducted a control experiment. The reaction system consists of 2,4‐DNP, Congo red Methylene Blue dye with NaBH_4_ in the absence of metallic catalyst Au/Fe_3_O_4_ composite microparticles. To determine the rate constant, the change of absorption peaks of 2,4‐DNP and CR at 358 nm, 496, and 664 nm are monitored at specific time intervals to create ln(C/C_0_) against the reaction time plot.^[47]^ Where C is the measured concentrations of 2,4‐DNP, CR, and MB pollutants at a particular time and C_0_ is the initial concentration, both derived from relative absorption measured at UV‐Vis spectroscopy. As we can see from Figure 4a, absorption of 2,4‐DNP decreased from 1.36 to 1.02 suggesting reduction reaction is relatively slow in the absence of a metallic catalyst. As shown in Figure 4d rate constant was found to be 7.6×10^−4^ s^−1^ for the reduction of 2,4‐DNP. Even more, as illustrated in Figure 4b, it is clear that the degradation process of Congo red dye is slower compared to 2,4‐DNP, and its absorption of Congo red dye decreased from 1.42 to 1.26. The determined rate constant, from Figure 4d, was about 4.9×10^−5^ s^−1^. In addition, the degradation of Methylene blue organic pollutant is relatively slower and its absorption changed from 0.82 to 0.78 within 130 seconds and the reaction constant derived from the pseudo‐first‐order kinetic model was 2.1×10^−4^ s^−1^. Hence, we assumed that the degradation reaction almost not occur without a catalyst. Therefore, we started to determine each degradation reaction in the presence of the Au/Fe_3_O_4_ composite micro catalyst.

**Figure 5:**
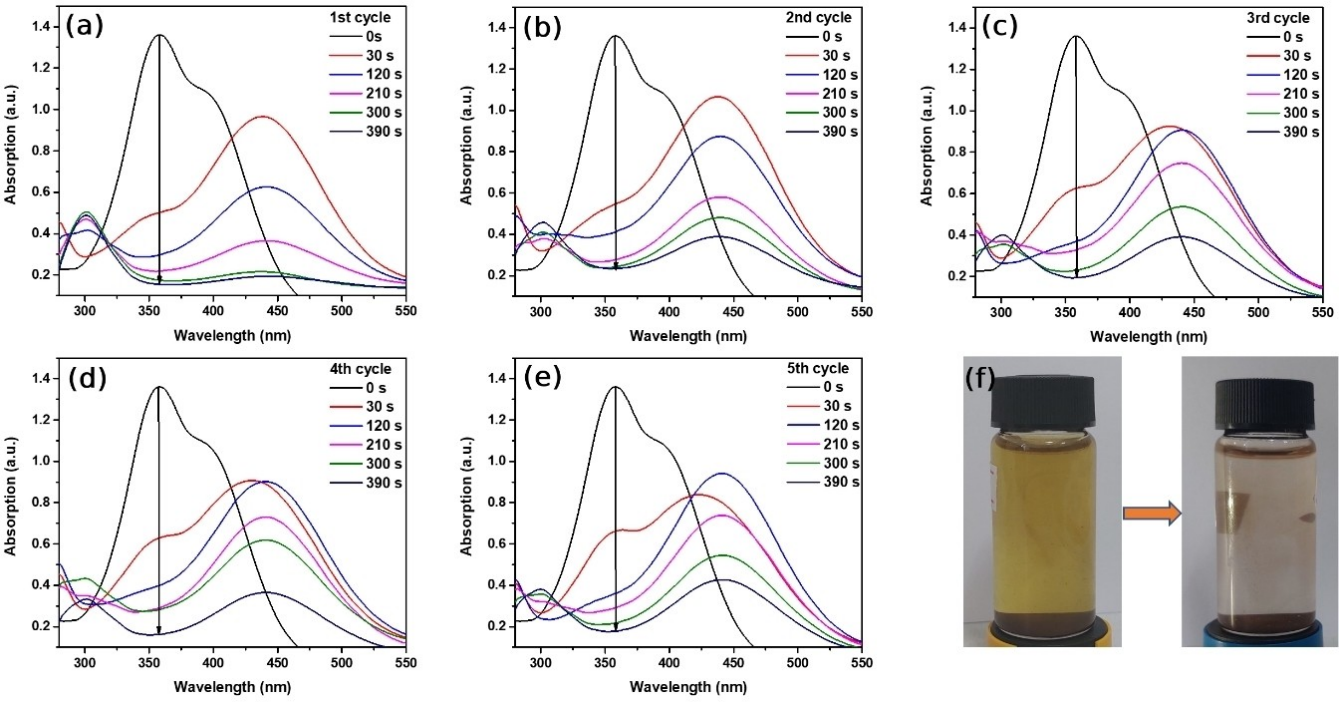
(a–e) Time‐dependent UV‐Vis absorptions of reduction reaction of 2,4‐DNP by NaBH_4_ in the presence of Au/Fe_3_O_4_ composite microparticles, (f) color of the solution before and after the catalytic reaction.

To understand the catalytic activity of Au/Fe_3_O_4_ composite microparticles, we monitored the time‐dependent characteristic UV‐Vis absorption peak of 2,4‐DNP which is located at 358 nm, and took the image of the color change of solution as displayed in Figure 5. Absorption at 358 nm relating to 2 electrons of oxygen atoms conjugated to a benzol ring with π‐bond decreased rapidly after the instant addition of an Au/Fe_3_O_4_ composite micro catalyst.^[48]^ Consecutive 5 successful cycles within the 390 seconds are represented in Figure 5a–e. Additionally, a new peak around 300 nm has started to appear, clearly associated with 2,4‐diaminophenol as peaks of 2,4‐DNP reduced. The color of the solution of 2,4‐DNP which was yellow became colorless indicating both the reduction of 2,4‐DNP occurring and also the formation of 2,4‐diaminophenol (2,4‐DAP), shown in Figure 5f.

**Figure 6:**
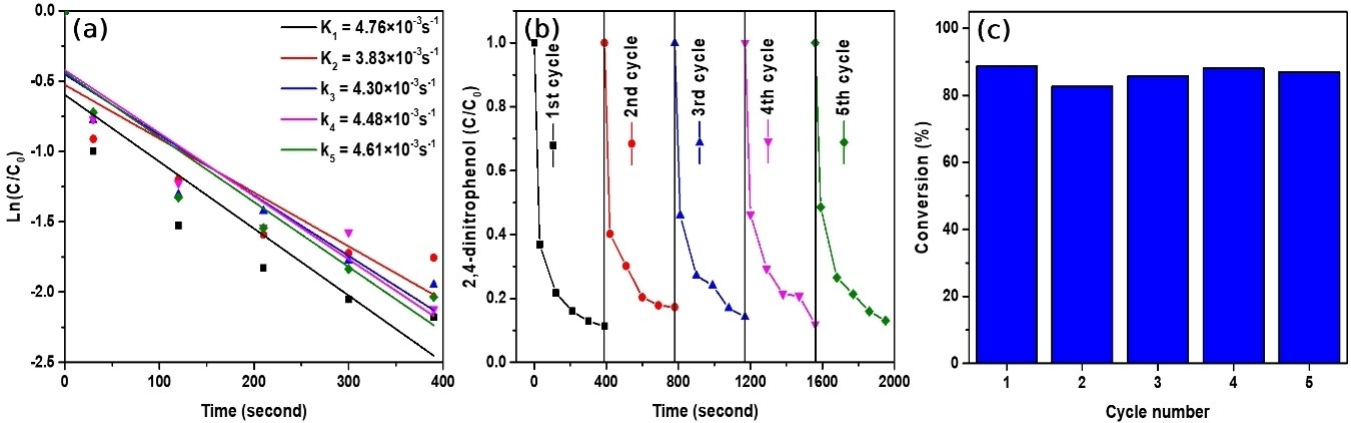
(a) First‐order kinetics plot of reduction of 2,4‐DNP in the presence of the Au/Fe_3_O_4_ composite microparticles. (b) The time‐dependent concentration of remaining 2,4‐DNP, (c) reusability of Au/Fe_3_O_4_ composite microparticles within 5 cycles.

To investigate the cyclic performance and reusability of Au/Fe_3_O_4_ composite microparticles, first‐order kinetic plots of each cycle were illustrated in Figure 6a. During the catalytic reaction, an excessive amount of reducing agent NaBH_4_ is used, therefore, we can assume that the reduction of 2,4‐DNP, degradation of CR, and MB process follow pseudo‐first‐order kinetics. As seen from the reaction kinetic plots, after 5 successful reductions of 2,4‐DNP in the presence of Au/Fe_3_O_4_ composite microparticles, the reaction rate constant remained almost the same as its initial reduction reaction which indicates the reaction rate constant has not been changed. Figure 6b shows the time‐dependent concentration of 2,4‐DNP. It can be seen that after the addition of Au/Fe_3_O_4_ composite micro‐catalyst, the initial concentration of 2,4‐DNP commenced to rapidly decrease to 0.1, and as a consequence, 2,4‐DAP has formed in the solution. Each cycle finished within only 390 seconds and showed a downward trend in the given period. The reusing ability of Au/Fe_3_O_4_ composite microparticles is shown in Figure 6c, and it suggests that our sample can be successfully reused for 5 cycles without any significant conversion decrement. Additionally, the conversion percentage of Au/Fe_3_O_4_ composite microparticles was preserved in the final reaction. Reduced conversion percentage of Au/Fe_3_O_4_ composite microparticles on the second cycle might be associated with the adsorbed reducing agent, 2,4‐DNP, and 2,4‐DAP molecules on the surface of the micro‐particle that has not been washed thoroughly. Therefore, some of the Au/Fe_3_O_4_ composite microparticles were aggregated resulting in the reduced rate constant and conversion percentage. Aside from that, Au/Fe_3_O_4_ composite microparticles preserved notable catalytic activity after 5 reduction reactions, its conversion percentage ranging from 88.67 % to 86.92 %, suggesting it possesses the potential to be utilized in the reduction of 2,4‐DNP as a reusable catalyst.

**Figure 7:**
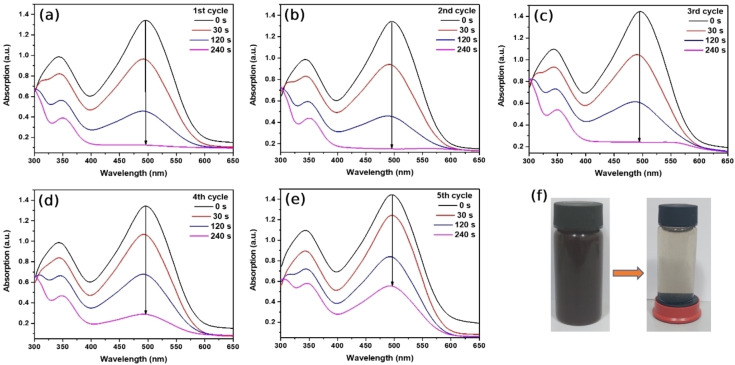
Time‐dependent UV‐Vis absorptions of the degradation process of CR by NaBH_4_ in the presence of Au/Fe_3_O_4_ composite microparticles.

Absorption spectra of 5 cycles are measured (Figure 7 a‐e) for the investigation of the catalytic reaction of degradation of CR reduced by the NaBH_4_ in the presence of the Au/Fe_3_O_4_ composite microparticles. The absorption peak located at 498 nm, associated with the electron transition of (π →π *) of the azo group, started to decrease quickly after injection of Au/Fe_3_O_4_ composite micro‐catalyst.^[49]^ After 240 seconds, the characteristic absorption peak of CR vanished and the color of the solution changed from red to transparent representing the successful catalytic reaction. Au/Fe_3_O_4_ composite microparticles possessed significant catalytic activity, however, after the second cycle, the reaction rate constant was lowered compared to the first reaction.

**Figure 8:**
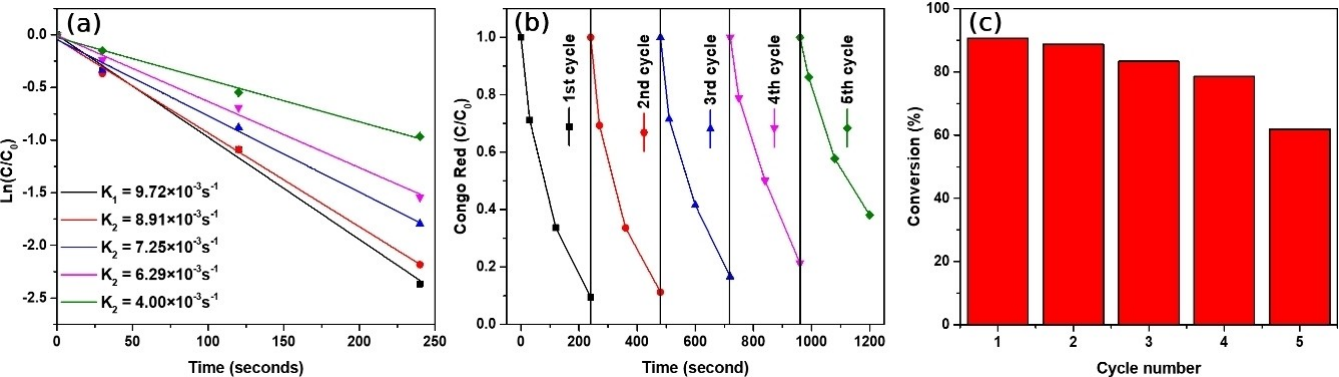
(a) First‐order kinetics plot of degradation of CR in the presence of the Au/Fe_3_O_4_ composite microparticles. (b) The time‐dependent concentration of remaining CR, (c) reusability of Au/Fe_3_O_4_ composite microparticles within 5 cycles.

Determined reaction rate constants of consecutive 5 degradation reactions of CR in the presence of Au/Fe_3_O_4_ composite microparticles are depicted in Figure 8a. The initial reaction rate constant was found to be 9.72×10^−3^ s^−1^ and it started to decrease in subsequent cycles. In contrast, the final reaction rate constant which was the 5th cycle became 4×10^−3^ s^−1^ suggesting that benzidine molecules produced from CR by degradation and a high amount of strong reducing agents are responsible for the aggregation of Au/Fe_3_O_4_ composite microparticles. After the degradation of CR, Au/Fe_3_O_4_ composite microparticles are highly aggregated which leads to weakened magnetic properties and lowered reactive surface area. These phenomena were the main reason for the reduced catalytic activity of Au/Fe_3_O_4_ composite microparticles. The proportion of unreacted CR in the solution has been monitored and shown in Figure 8b. In the first utilization of Au/Fe_3_O_4_ composite microparticles, the concentration of CR dye is reduced to 0.1 indicating successful degradation. However, after 5 cycles, Au/Fe_3_O_4_ composite microparticle's catalytic activity decreased and the proportion of unreacted CR reached around 0.4 within 240 seconds. For the reusability of Au/Fe_3_O_4_ composite microparticles, in Figure 8c, only 60 % of the catalytic activity of Au/Fe_3_O_4_ composite microparticles was preserved in the last reaction due to poor dispersity caused by the high number of aggregated particles.

**Figure 9:**
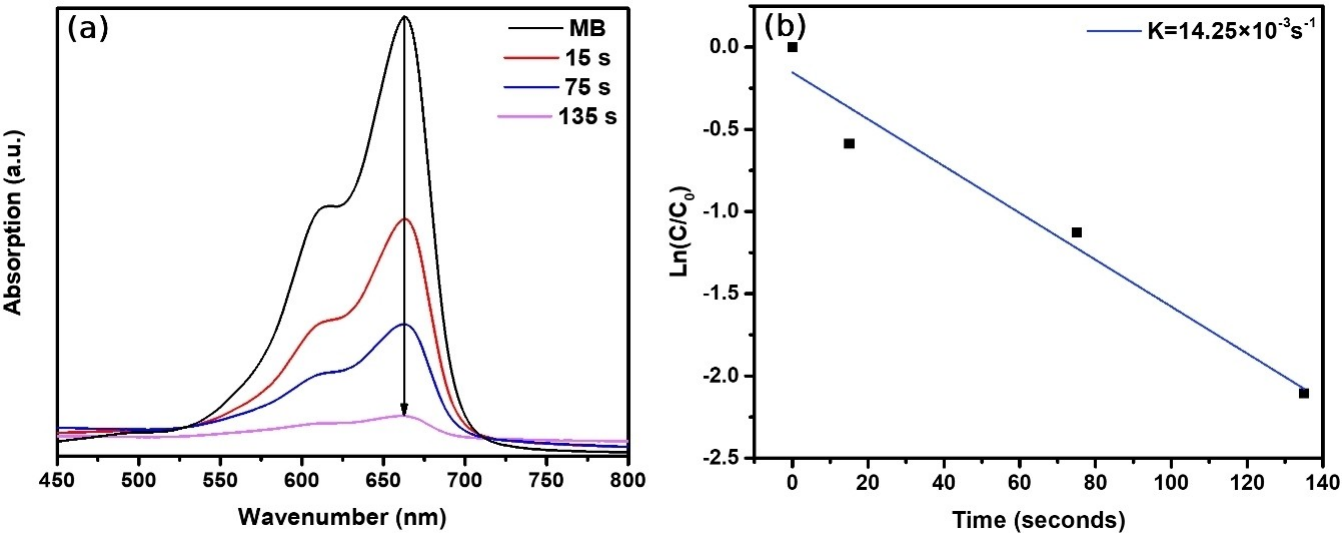
(a) Time‐dependent UV‐Vis absorptions of the degradation process of MB by NaBH_4_, (b) First‐order kinetics plot of degradation of MB in the presence of Au/Fe_3_O_4_ composite microparticles.

As shown in Figure 9a, the catalytic activity of Au/Fe_3_O_4_ composite microparticles was examined on the clock reaction of MB to leucomethylene blue (LMB) reduced by NaBH_4_. When the blue‐colored solution of MB is reduced, its maximum absorption of 665 nm associated with n–π* electron transitions of the MB dye will decrease then the solution is decolorized which is a clear indication of the formation of LMB.^[50]^ Once Au/Fe_3_O_4_ composite microparticles were added to the MB solution in the presence of NaBH_4_, it rapidly became colorless. Decrement of the absorption peak at 665 nm was caused by the break of p conjugation in MB molecules. All of the catalytic reactions such as the reduction of 2,4‐DNP, CR, and MB can be explained by electron transfer from donor BH^4−^ ions to acceptor organic compounds. Au/Fe_3_O_4_ composite microparticles were responsible for playing the role of an acceptor and a donor of electrons and lowering the activation energy of the reduction process, making it the catalyst for redox reaction.^[49]^ The reaction rate constant of Au/Fe_3_O_4_ composite microparticles for degradation of MB was determined to be 14.25×10^−3^ s^−1^ from the first‐order kinetic plot (Figure 9b). In other words, Au/Fe_3_O_4_ composite microparticles possess great catalytic activity for the degradation of MB. However, as aforementioned in zeta potential measurements, Au/Fe_3_O_4_ composite microparticles have a strong negative charge on the surface, therefore, we are predicting that strong electrostatic interaction between positively charged MB molecules and negatively charged Au/Fe_3_O_4_ composite microparticles leading to huge aggregation. Once Au/Fe_3_O_4_ composite microparticles aggregated, they lost magnetic property and reusability as a catalyst. For this reason, our result suggests Au/Fe_3_O_4_ composite microparticles are not potentially reusable for the degradation of MB dye due to strong electrostatic interaction.

In order to evaluate the catalytic activity of Au/Fe_3_O_4_ composite microparticles, normalized reaction rate constants were calculated. K_nor_ is a crucial parameter for understanding the catalytic capability of Au/Fe_3_O_4_ composite microparticles, therefore, we explored the relationship between the reaction rate constant and the amount of Au/Fe_3_O_4_ composite microparticles, summarized in Table S1. Normalized reaction rate constants were obtained according to K_nor_=K_app_/m, where K_nor_ is the normalized reaction constant rate, K_app_ is the apparent constant rate and m is the mass of the catalyst.^[51]^ To disclose the future application of the Au/Fe_3_O_4_ composite microparticles, obtained K_nor_ was compared with other literature in Table 2. It can be seen that our prepared Au/Fe_3_O_4_ composite microparticles exhibited excellent initial catalytic activity compared to recent works. However, the reusability of Au/Fe_3_O_4_ composite microparticles was decreased after the first reaction on reduction of CR and MB organic pollutant, while Au/Fe_3_O_4_ composite microparticles preserved its first catalytic activity for reduction of 2,4‐DNP. Therefore, we evaluate Au/Fe_3_O_4_ composite microparticles as promising reusable catalysts with high surface area for reduction of 2,4‐DNP.

**Table 2:** Comparison of previously reported values K_nor_ of Au/Fe_3_O_4_ composite materials.

Structure of catalyst	Organic pollutant	Particle diameter (nm)	K_app_ (10^−3^ s^−1^)	K_nor_ (s^−1^mg^−1^)	Cycling efficiency	Reference
Au_0.3_Pd_0.7_@BGNs/Fe_3_O_4_	4‐NP	Au‐Pd alloy 2.65	165.1	17.990	9 cycles	[52]
Au‐Ag‐γ‐Fe_2_O_3_/rGO	4‐NP	5.3	13.3	0.443	5 cycles 95 %	[53]
Au/mSiO_2_@RGO	4‐NP	600–2400	15.0	0.037	10 cycles 100 %	[54]
Au/Fe_3_O_4_	2,4‐DNP	1700	4.7	1.044	5 cycles 86.2 %	(this work)
Au/AC	CR	AuNP 14.9	0.11	0.012	–	[55]
Au@Pd@RuNP	CR	110	24.90	–	–	[56]
Au3‐Cu1/rGO	CR	Au‐Cu 15	12	120	–	[57]
Au/Fe_3_O_4_	CR	1700	9.7	2.155	5 cycles 60 %	(this work)
Au/mSiO_2_@RGO	MB	600‐2400	12.1	0.030	–	[54]
Au@PPy/Fe_3_O_4_	MB	500	4.4	2.660	5 cycles nearly 100 %	[58]
Fe_3_O_4_@Tannic Acid@Au	MB	20	9.1	0.004	5 cycles >90 %	[59]
Au/Fe_3_O_4_	MB	1700	14.25	3.166	–	(this work)

## 3 Conclusions

In this work, we prepared Au/Fe_3_O_4_ composite microparticles using the co‐precipitation method for Fe_3_O_4_ microparticles and chemical reduction method for gold nanoparticles reduced and stabilized by citrate ions on the surface of the Fe_3_O_4_ particles. Au/Fe_3_O_4_ composite microparticles possessed great magnetic separation properties and also reusing ability for reduction of 2,4‐DNP and CR in the presence of strong reducing agent NaBH_4_. Our prepared samples exhibited high crystallization in the diffraction pattern, enhanced light absorption in the NIR region, and negative charge on the surface proven by XRD, Zeta potential, and UV‐Vis spectroscopy characterizations. The mean diameter of synthesized Au/Fe_3_O_4_ composite microparticles was determined to be about 1.7 μm, while the gold nanoparticle located on the surface of the Fe_3_O_4_ particle was found to be around 210 nm from the SEM image. The most notably, Au/Fe_3_O_4_ composite microparticles showed a reaction rate constant of 4.76×10^−3^ s^−1^ for the reduction of 2,4‐DNP. Also, it preserved almost the same even after 5 cycles which were 4.61×10^−3^ s^−1^ and conversion percentage became 89.62 % suggesting it has a potential ability to reuse in reduction of 2.4‐DNP by magnetic separation. In comparison, Au/Fe_3_O_4_ composite microparticles dropped their conversion effect to 61.86 % due to poor dispersity induced by benzidine molecules produced from the degradation of CR and strong reducing agent. In contrast, our results suggest that Au/Fe_3_O_4_ composite microparticles are vulnerable for degradation of MB because of strong interaction between positively charged MB molecules and negatively charged Au/Fe_3_O_4_ microparticles. Thus, the increased aggregation resulted in weakened magnetic property and reduced active surface area during the reduction process. This study suggests that using the Au/Fe_3_O_4_ composite microparticles for the reduction of aromatic compounds with nitro groups is the most suitable.

## Conflict of Interests

The authors declare no conflict of interest.

4

## Supporting information

As a service to our authors and readers, this journal provides supporting information supplied by the authors. Such materials are peer reviewed and may be re‐organized for online delivery, but are not copy‐edited or typeset. Technical support issues arising from supporting information (other than missing files) should be addressed to the authors.

Supporting Information

## Data Availability

The data that support the findings of this study are available from the corresponding author upon reasonable request.
