# Supplementary material for: Comparative Study of Catalytic Activity of Recyclable Au/Fe3O4 Microparticles for Reduction Of 2,4‐Dinitrophenol and Anionic, Cationic Azo Dyes
Source: ChemistryOpen. 2024 Apr 16;13(9):e202300297. doi: 10.1002/open.202300297 (PMC11633347; doi:10.1002/open.202300297)
Supplement: Supplementary file 1 — Supporting Information [file OPEN-13-e202300297-s001.pdf]

ChemistryOpen

Supporting Information

Comparative Study of Catalytic Activity of Recyclable Au/Fe_3O_4 Microparticles for Reduction Of 2,4-Dinitrophenol and Anionic, Cationic Azo Dyes.

Ikhbayar Batsukh, Tegshjargal Khishigjargal, Uuriintuya Dembereldorj, Munkhtsetseg Sambuu, Erdene-Ochir Ganbold,* and Erdene Norov

Table S1. Normalized reaction rate constant K_{nor} of consecutive 5 cycles of Au/Fe_3O_4 composite microparticle for degradation of 2,4-DNP, CR, and MB.

Cycle number	K_{nor} for 2,4-DNP ($s^{-1}mg^{-1}$)	K_{nor} for CR ($s^{-1}mg^{-1}$)	K_{nor} for MB ($s^{-1}mg^{-1}$)
1st cycle	1.044	2.155	3.166
2nd cycle	0.851	1.980	-
3rd cycle	0.955	1.611	-
4th cycle	0.995	1.397	-
5th cycle	1.024	0.888	-
